# Dual mobility cup reduces dislocation rate after arthroplasty for femoral neck fracture

**DOI:** 10.1186/1471-2474-11-175

**Published:** 2010-08-06

**Authors:** Sarunas Tarasevicius, Mantas Busevicius, Otto Robertsson, Hans Wingstrand

**Affiliations:** 1 Department of Orthopedics, Lund University Hospital, Getingevagen 4 Lund 22185 Sweden; 2 Department of Orthopedics, Kaunas Medical University, Eiveniu 2, Kaunas,LT-50009, Lithuania

## Abstract

**Background:**

Hip dislocation after arthroplasty for femoral neck fractures remains a serious complication. The aim of our study was to investigate the dislocation rate in acute femoral neck fracture patients operated with a posterior approach with cemented conventional or dual articulation acetabular components.

**Methods:**

We compared the dislocation rate in 56 consecutive patients operated with conventional (single mobility) cemented acetabular components to that in 42 consecutive patients operated with dual articulation acetabular components. All the patients were operated via posterior approach and were followed up to one year postoperatively.

**Results:**

There were 8 dislocations in the 56 patients having conventional components as compared to no dislocations in those 42 having dual articulation components (p = 0.01). The groups were similar with respect to age and gender distribution.

**Conclusions:**

We conclude that the use of a cemented dual articulation acetabular component significantly reduces the dislocation rates in femoral neck fracture patients operated via posterior approach.

## Background

Dislocations of hip prosthesis in femoral neck fracture patients remain a serious problem. A metaanalysis [1] revealed a median dislocation rate of 10,7% in femoral neck fracture patients treated with THA (total hip arthroplasty), five time higher as compared to arthroplasty for osteoarthritis. Possible explanations may be a greater tendency to fall, less muscular control, and increased ligament laxity in hip fracture patients [1,2]. However, the dislocation rate is also affected by other factors directly related to the surgical procedure; e.g. surgical approach, choice of implant, and orientation of the acetabular and femoral components. Morrey [3] reported that the dislocation rate was 2 to 3 times greater after primary THA with the posterior approach as compared to THA with an anterior approach. Enocson et al. [4] investigated factors influencing the stability of the prosthetic hip in femoral neck fracture patients and reported that the anterolateral approach was associated with a lower risk of dislocation than the posterolateral approach. Despite the reported greater dislocation rate with posterior approach it is still widely used. Therefore, surgeons using this approach in patients with a high risk of dislocation might consider selecting a more suitable implant. It has been reported that using a greater femoral head size or bipolar acetabular components reduces the risk of dislocation [5]. Recently, dual articulation acetabular systems have been reported to provide better implant stability as well as good long term results [6].

We are not aware of any reports on the use of dual articulation acetabular components in femoral neck fractures. Thus, the aim of our study was to investigate the dislocation rate in femoral neck fracture patients treated with conventional or dual articulation acetabular components.

## Methods

Our study concerns patients treated for femoral neck fracture in the Kaunas Medical University Hospital from July 2004 to July 2008. 128 patients were treated during this period of which 116 had a total arthroplasty (THA). Twelve patients treated with hemiarthroplasty were excluded.

During the first two years all 63 patients were treated with THA and no dual articulation cups (DAC) were used (Table 1). In July 2006 a DAC implant was introduced and used in all 53 femoral neck fractures patients until the end of the study in July 2008. All the DAC cups (Avantage (Biomet) were cemented and in all a cemented WLink femoral component with a 28 mm femoral head was used.

**Table 1:** Implant related data in the conventionally cemented cup group (n = 63)

Implant type	Number of patients
C-stem with 28 mm femoral head	7

Aesculap bicontact with 28 mm femoral head	21

WLink with 32 mm femoral head	31

ScanHip Classic II with 28 mm femoral head	4

During the four year period, all THA were performed by one of 4 experienced orthopedic surgeons.

All patients were operated via posterior approach without posterior soft tissue repair with either spinal or epidural anesthesia, and all patients received the same rehabilitation program.

Both groups of patients were followed for a period of one year after surgery and the dislocation rate was registered. 23 patients died during the first year after surgery (12 in the conventional THA group and 11 in the dual articulation group) none of which dislocated and they were excluded from further analysis.

T-test was used to compare the differences in age between the groups. Chi square test was used to compare proportions of dislocations and gender between groups. A p-value of < 0.05 was considered significant. We used the SPSS software.

The study was approved by the ethical committee of the Kaunas Medical University Clinics (BE-2-8).

## Results

One year after surgery 98 patients were alive. The mean age and gender proportions did not significantly differ between the groups (table 2). Eight dislocations had occurred in the 56 patients with conventional cemented cups whereas no dislocations were observed in the group of 42 in which the DAC (Avantage cup) was used (p = 0.01). None of the dislocations were associated with significant trauma. Four patients experienced more than one dislocation during the first year, three of these patients had a cup revision to a DAC. The information regarding dislocations and revisions is presented in table 3. The analysis of dislocations in respect to the femoral head used in conventional THA groups showed that increasing head size was associated with decreasing number of dislocations. Compared to no dislocations among the 42 DAC, there were 6 dislocations out of the 32 THA hips with 28 mm femoral heads (p = 0.005) and 2 dislocations out of 31 THA with 32 mm femoral head (p = 0.18).

**Table 2:** Choice of cup, age and sex distribution in patients alive one year after surgery.

Variables	Age	Gender (M-male, F-female)
Conventional cup, n = 56	74 (SD 10)	M-23, F-33

DAC group, n = 42	75 (SD 10)	M-11, F-33

p	0.6	0.14

**Table 3:** Dislocated THA in the conventional cup group.

Patient's number	Implant type	Number of dislocations	Revision surgery
**1**	Aesculap Bicontact, 28 mm	2	Yes

**2**	Aesculap Bicontact 28 mm	3	No

**3**	Aesculap Bicontact 28 mm	2	Yes

**4**	Aesculap Bicontact 28 mm	3	Yes

**5**	ScanHip 28 mm	1	No

**6**	ScanHip 28 mm	1	Yes

**7**	WLink 32 mm	1	No

**8**	WLink 32 mm	1	No

## Discussion

Within one year of surgery, 8 out of 56 hips with a conventional cemented cup had dislocated. Considering the known high dislocation rate after THA for femoral neck fracture [1] it might be considered either not to use a posterior approach or select an implant less prone to dislocate. With the DAC we had no dislocations. As with DAC in our material, it has been reported that bipolar hip prostheses significantly reduce dislocation rates as compared to conventional THA [5], and they are widely used for treatment of femoral neck fractures [7]. However, bipolar implants have been associated with migration into the acetabulum and acetabular osteolysis [8].

In our study, all the THA were performed by one of 4 experienced orthopedic surgeons. Although 4 different THA systems were used in the conventional group and two different head sizes, these implants had been in use for a long period before the study was started, thus they were familiar to the surgeons, and our dislocation rate was similar to that reported in the literature [1]. Furthermore, no dislocations occurred after the introduction of DAC in spite of a possible "learning curve" with the introduction of a new implant.

That both 28 mm and 32 mm heads were used in the conventional THA group may be regarded as a weakness of the paper. Larger head size in THA has been associated with decreasing dislocation rates [9] which is in concordance with our material in which the dislocation for 28 mm femoral heads was 3 times higher than for 32 mm heads. However, some surgeons may be reluctant to opt for larger head sizes considering that they have been associated with higher polyethylene wear and increased risk of aseptic loosening [10-12].

The economy when choosing an implant is of great importance. The Dual articulation Avantage cup is approximately three times more expensive than a conventional cemented cup. In 56 femoral neck fracture patients we had 14 episodes of prosthetic dislocation in 8 patients within one year after surgery with associated additional costs, and there were 3 revisions due to recurrent dislocations. Adding to the cost of surgery there were additional costs for prolonged hospital stay as well as postoperative rehabilitation. Consequently the initial savings using a conventional implant, at least one with 28 mm head, may be questionable not considering the pain and suffering of the patients involved.

## Conclusions

We found that changing from conventional THA, using a mixture of 28 mm and 32 mm heads, to a dual articular acetabular component arthroplasty significantly reduced the dislocation rate, mainly due to a higher risk of dislocation in the 28 mm group.

## Competing interests

The authors declare that they have no competing interests.

## Authors' contributions

ST: data collection, compilation and analysis, writing manuscript, MB: data collection, editing manuscript, OR: statistical analysis, editing manuscript, HW: organizing study, editing manuscript.

All authors read and approved the final manuscript.

## Pre-publication history

The pre-publication history for this paper can be accessed here:

http://www.biomedcentral.com/1471-2474/11/175/prepub
